# Evaluation of Sexual Communication Message Strategies

**DOI:** 10.1186/1742-4755-8-15

**Published:** 2011-05-20

**Authors:** W Douglas Evans, Kevin C Davis, Cindy Umanzor, Kajal Patel, Munziba Khan

**Affiliations:** 1 The George Washington University, School of Public Health and Health Services, Department of Prevention and Community Health, 2175 K Street, NW, Suite 700, Washington, DC, 20037 USA; 2 Research Triangle Institute, Social, Statistical and Environmental Sciences, Public Health Economics and Policy Research Program, 3040 East Cornwallis Road, Research Triangle Park, NC, 27709 USA

## Abstract

Parent-child communication about sex is an important proximal reproductive health outcome. But while campaigns to promote it such as the Parents Speak Up National Campaign (PSUNC) have been effective, little is known about how messages influence parental cognitions and behavior. This study examines which message features explain responses to sexual communication messages.

We content analyzed 4 PSUNC ads to identify specific, measurable message and advertising execution features. We then develop quantitative measures of those features, including message strategies, marketing strategies, and voice and other stylistic features, and merged the resulting data into a dataset drawn from a national media tracking survey of the campaign. Finally, we conducted multivariable logistic regression models to identify relationships between message content and ad reactions/receptivity, and between ad reactions/receptivity and parents' cognitions related to sexual communication included in the campaign's conceptual model.

We found that overall parents were highly receptive to the PSUNC ads. We did not find significant associations between message content and ad reactions/receptivity. However, we found that reactions/receptivity to specific PSUNC ads were associated with increased norms, self-efficacy, short- and long-term expectations about parent-child sexual communication, as theorized in the conceptual model.

This study extends previous research and methods to analyze message content and reactions/receptivity. The results confirm and extend previous PSUNC campaign evaluation and provide further evidence for the conceptual model. Future research should examine additional message content features and the effects of reactions/receptivity.

## Introduction

Parent-child sexual communication is an important proximal outcome for programs designed to reduce negative adolescent reproductive health outcomes such as early sexual activity, HIV/STIs, and pregnancy or repeat pregnancy [1-3]. The Parents Speak Up National Campaign (PSUNC) is an evidence-based social marketing campaign that has been shown to be efficacious in increasing parent-child communication and behavioral determinants such as beliefs and intentions to communicate [4,5]. The campaign's conceptual model, based on Social Cognitive Theory, has also been validated [6]. Evans and colleagues (2011) found that increased parent-child communication is mediated by higher parental outcome expectations and that such communication successfully reduces sexual risk behaviors in the child [7].

However, while parent-child communication is an important outcome, and campaigns to promote it such as PSUNC have been effective, little is known about how messages influence parental cognitions and behavior. Previous studies have shown that constructs drawn from messaging theory explain adoption of health behaviors and avoidance of risk behaviors promoted by media campaigns [8-10]. Specifically, sensation value [11,12], message themes, advertising stylistic features, and respondent characteristics can explain cognitive and behavior response to health messages [9,10]. What message features explain responses to sexual communication messages?

The purpose of this study is to compare the strength of associations between exposure to individual PSUNC advertising executions and parent-child sexual communication. Identifying these associations is an important step toward understanding how social marketing and message strategies work. Gaining a deeper understanding of mechanisms underlying these strategies will help social marketers develop improved campaign messages and potentially achieve greater changes in cognitive and behavioral outcomes in future. The strength of associations between PSUNC advertising and parent-child sexual communication may be mediated by specific message features found to be significant in previous research.

### Previous message receptivity research

A number of studies in health communication have established measures of "receptivity" to public service advertising (PSA) that capture audiences' subjective appraisals of message persuasiveness, believability, and other aspects of cognitive processing [13,14]. These measures are often used as proxies of potential ad effectiveness as they have been shown to predict changes in attitudes towards the social issues and subject matter of PSAs these messages were developed for. Such measures can be used to assess effectiveness of campaign messages during development and prior to implementation.

Two related theories, the Activation Model of Information Exposure (AMIE) [15], and the Limited Capacity Model of Mediated Message Processing (LCM) [16], further propose a variety of stylistic message components that promote audience receptivity to messages. This research, in turn, has been used in the design of message testing research and development of measures of message recall, receptivity, and response.

Studies based on the LCM have explored the effects of specific visual, audio, and format features on attention and processing [16,17]. Other studies examine the effects of specific message features on message recall using population surveys [17,18]. Studies based on AMIE examine attention to a message (eg, a drug-use prevention television advertisement aimed at adolescents) as a function of and an individual's need for sensation (NFS, also called sensation seeking) and the level of stimulation provided by the message. Youth with high NFS are more likely to report health risk behaviors such as drug use (Palmgreen, Lorch, Stephenson, et al, 2007) [11], cigarette smoking [18], and risky sex [19].

There have been some criticisms of these approaches in recent studies, and the field has recently advanced through testing of AMIE and LCM propositions in the context of large-scale anti-tobacco media campaigns [9,10]. These studies have developed methods for evaluating the message strategies and approach of public health advertising, which we have followed and built upon in the current study. For example, Davis and colleagues (2010) found that tobacco control ads based on a 'Why to Quit' (or reasons why cessation would be beneficial) message theme were perceived as more effective and associated with more positive smoking cessation attitudes than 'How to Quit' or messages attacking the tobacco industry [10]. In the present research, we build on these methods and apply them to advertising aimed at promoting parent-child sexual communication.

### PSUNC social marketing and messaging

As described elsewhere, the Parents Speak Up National Campaign (PSUNC) was implemented by the US Department of Health and Human Services with the objective of improving the frequency, quality, and timing of parent-child sexual communication [4]. PSUNC was formally launched on June 21, 2007, using paid and unpaid television, radio, print, and outdoor advertisements. The primary communication channel for the campaign is TV and radio public service announcements (PSAs) designed for a general audience and targeted PSAs for African American and Hispanic audiences. Additionally, there were static Web-page designs and print-based PSA formats. The PSAs used actors and settings specific to the racial/ethnic audience but retained the same specific messages and script. Health messaging specific to a target audience is more salient and more likely to be recalled [20]. The PSAs feature age-appropriate youth letting their parents know that it is okay to talk to them about sex and urging them to "talk early and often" [21].

Additionally, PSUNC included the http://4Parents.gov Web site, community outreach and training, and associated parent and adolescent guides. The public awareness campaign was designed to drive traffic to this Web site.

The campaign as a whole was based on social cognitive theory (SCT) and hypothesized increased parent-child communication would result from modeling effective communication behavior and promoting positive outcome expectancies [6]. PSUNC develops a credible and appealing argument for delaying initiation of sexual activity by communicating personal (social, educational, career-related) advantages of delayed sexual debut [8]. The campaign also promotes parents' self-efficacy and outcome-efficacy to communicate the health risks of early sexual debut and benefits of communication with their children.

In the campaign's behavior change model, published elsewhere, these factors are hypothesized as mediators of campaign effects on parent-child sexual communication [21]. Grounded in SCT, the behavior change model posits that increased parent-child communication will result from social modeling of effective communication behavior and promotion of positive outcome expectancies [4,6]. The PSUNC behavior change model posits that these constructs, including perceived norms of teen sexual activity and outcome expectations about the effectiveness of parent-child communication in delaying sexual debut, mediate parent-child communication outcomes [21].

Following the behavior change model, the PSUNC evaluation was designed using constructs drawn from SCT. Prior evaluation studies have shown the PSUNC behavior change model to be valid in predicting the mechanisms of change between exposure to PSUNC messages and adoption of the aforementioned cognitions related to parent-child communication [5,7]. However, there is less understanding about how parents' receptivity to parent-child communication messages enhances these processes above the effects of simple exposure to those messages. Understanding social cognitive precursors provides information that can be used to refine and improve messaging and social marketing campaigns. Although many studies have examined the role of social cognitive factors in behavior change, the current study joins the small but important set of studies that have examined the cognitive factors that precede parent-child communication outcomes [22-24]. In this study, we examine what aspects of message content, theme, and audience receptivity to those messages that are associated with adoption of specific social cognitions among parents exposed to sexual communication messaging.

Early PSUNC advertising focused on child-voiced messages. Since 2007, additional creative media have been developed for PSUNC that use parent-voiced messaging aimed at reducing self-imposed barriers to communication. For example, the "Mimes" television ad features a father and daughter (who are both mimes) portraying the difficulty, from the parent's point of view, in initiating parent-child communication. Another recently developed ad called "Gadget" focuses on parents' control, or lack thereof, of their child's exposure to sex but discusses ways that parents can influence their child's behavior through parent-child communication. These ads focus more on the parents' point of view in initiating parent-child communication. Other new media are also being developed for PSUNC, including a new ad that will focus on the http://4parents.gov Web site and promoting Web site use.

PSUNC ads also differ in terms of messaging strategies, including appeals to parents' personal sense of responsibility (i.e., it is their responsibility to talk with their children). and addressing common defenses or barriers to parent-child communication. The approach used in each of the ads examined in this study is described below.

### Current Study Objectives

The current study compares parents' reactions and receptivity to parent-voiced and child-voiced ads as well as compare reactions and receptivity across the two primary message strategies noted above. Specific aims of this study are to:

1. Content analyze ads and identify specific, measurable message and advertising execution features;

2. Develop quantitative measures of those features, including message strategies, marketing strategies, and voice and other stylistic features; and

3. Evaluate the relationship between a) message strategies/features and ad reactions/receptivity, and b) ad reactions/receptivity and parents' cognitions related to sexual communication.

With respect to aim 2), we conducted an in-depth content analysis and coding scheme to quantify features of 4 PSUNC advertisements and used results in subsequent multivariable logistic regression analyses. Parents' reactions and receptivity to PSUNC ads was measured with a single scale variable that was subjected to factor and reliability analysis. We first regressed PSUNC message reactions/receptivity on ad content. Using parents' cognition scale variables validated in previous research [5], we then regressed on message reactions/receptivity.

## Methods

### Study design and data collection

The present study was part of a larger evaluation of PSUNC that has been described in detail elsewhere [4]. Briefly, the evaluation included multiple evaluation surveys that were conducted using the Knowledge Networks (http://www.knowledgenetworks.com) Web-based consumer panel, which is drawn from a nationally representative sample of US households. Knowledge Networks panelists are first selected using random-digit-dialing (RDD) telephone methodology, providing a probability-based starting sample of US telephone households. The panel is adjusted to match US Census demographic benchmarks. Households that do not already have Internet connectivity are provided a Web TV set-top box and free monthly Internet access to ensure representation of non-Internet households.

Data for the present study come from a field-based media tracking survey of parents, also collected via online survey from the Knowledge Networks panel. This survey was administered in the fall of 2009 to assess actual (as opposed to controlled) parent exposure to PSUNC in the U.S. as well as parents' reactions and receptivity to PSUNC ads. The survey included a total of 1,804 parents of children 10-14 years old. Mothers (N = 1,253) were oversampled since it is well known that mothers more often engage in sexual communication with their children compared to fathers [25-27], and the PSUNC ads reflect this in their mother-centric messages.

Participants in the present study were parents (mothers and fathers were sampled separately) of children aged 10-14 years recruited from the Knowledge Networks panel. Participants completed a media tracking survey designed to evaluate exposure, reactions and outcomes of PSUNC. This PSUNC National Media Tracking Survey measured parents' awareness of and reactions to PSUNC ads in the real world and on a national level. Parents completed a 64-item survey online at the secure Knowledge Networks Web site. The survey consisted of questions on sociodemographic characteristics; knowledge, attitudes, and beliefs about parent-child communication; parent-child communication social norms and expectations; media habits; and other community-level influences, such as school-based abstinence programs. During the survey, participants were also shown video files of PSUNC ads and then asked how frequently they had seen those ads on television. This is a validated method of measuring encoded ad exposure [28]. After viewing the ads, participants were also asked a battery of questions about their reactions and receptivity to the ads (described below).

Our analysis focuses on the identification of main message themes and categorizes the use of various production features, actor characteristics, message channels, and music features in four PSUNC messages: (1) Mimes, (2) Gadget, (3) Muffin-head, (4) Talk-to-Me Ads. "Mimes" television ad features a father and daughter (who are both mimes) portraying the difficulty, from the parent's point of view, in initiating parent-child communication. "Gadget" focuses on parents' control, or lack thereof, of their child's exposure to sex but discusses ways that parents can influence their child's behavior through parent-child communication. The "Muffinhead" ad encourages parents to speak to their children about sex and reassures them that it won't negatively affect their relationship. The "Talk to Me" ad, on the other hand, attempts to parents' personal sense of responsibility. Overall, the four ads deal with addressing common defenses or barriers to parent-child communication.

All PSAs used in this experiment were developed for specific racial and ethnic target audiences. The PSAs targeted general audiences, African Americans, and Hispanic. African Americans and Hispanics receive their targeted versions of the materials, and all other racial/ethnic groups received materials for general audiences. Our analysis concentrates on content analysis of the four ad models and identify specific, measurable message and advertising execution characteristics; and develop quantitative measures of those characteristics, including message strategies, marketing strategies, and voice and stylistic features. Table 1 provides an overview of the ads and messaging.

**Table 1:** PSUNC advertisement message strategies

Objective	Target Audience	Strategy	Message	Title	Dominant Voice and POV of Execution
Encourage and educate parents on how to talk to their children in a natural and effective manner about waiting to have sex	Parents of children 11-14	Appeal to personal sense of responsibility	Parents, don't wait to talk to your kids	"Talk to Me"	Kids
		Address common defenses	Your relationship with your child will not be lost	"Muffin-head"	
			You can't always be in control	"Gadgets"	Parents
			Bringing up the subject won't trigger difficult situation.	"Mime"	

### Content analysis

As a first step in evaluating the effects of message content on message receptivity and reactions, we developed a codebook designed to identify main message themes and categorize the use of various production features, actor characteristics, message channels, and music features in various PSUNC. Two coders independently reviewed the ads and coded based on the following categories. They were instructed only to code for what was explicit in the advertisements. After they coded each ad, they compared results. In cases where there was disagreement, the lead author of this paper viewed the ads with them and together the group arrived at an agreed upon coding for category in question.

Based on previous research described earlier, we developed codes for 4 categories of advertising content: Message strategies, themes, stylistic features, and characters. For *message strategies*, we coded for 1 dominant strategy in terms of the appeal portrayed in the ad. The specific coding categories and values for each ad content analyzed in this study were as follows:

• Appeals to Personal Responsibility: Emphasizes that it is the parent's personal responsibility for communicating with their child about sex.

• Addresses Common Barriers: Depicts common barriers to parent-child communication.

For *message themes*, there were 4 coding categories and ads could score from 0 to 4 themes. Thus each ad would have a theme score of up to 4. The categories and values were as follows:

• Don't Wait to Talk: Portrays the child's desire for parents to talk to them.

• Relationship With Child: Reassures the parent that their relationship with their child will not be lost by talking to their child about sex.

• Can't Always Be in Control: Depicts the fact that kids are exposed to sexual messages from many sources including TV, music and the internet.

• Communication Need Not be Embarrassing or Difficult: Emphasizes to the parent that talking with their child about sex may seem embarrassing or difficult to bring up.

For *stylistic features*, the categories and values we examined a number of visual and audio characteristics and added them together. The categories were as follows:

• Edits: the number of cuts or edits in the ad (a cut/edit occurs whenever the camera changes from one shot to another, including cuts to text screens, and cut to sponsor)

• Unrelated Cuts: the number of transitions to a new physical environment or "scene" within the ad (i.e., an environment that is not visible in, or contiguous with, the previous shot).

• Statistics: Does the advertisement use any statistics?

• Humor/Parody: The advertisement uses humor or imitations/comparisons to depict parents' difficulty in communicating with their child about sex.

• Special Effects/Graphics: Does the ad feature any special effects or graphics interacting with people in the ad?

• Acted Out: The message(s) of the ad are portrayed visually or "acted out" versus simple delivery of information.

• Point of View: What is the dominant point of view or "voice" of the ad (parent or child)?

• Music: the use of music throughout the ad

• Tempo: the tempo, or speed, of the music in the ad

• Volume: the volume of the music in the ad, relative to talking and other sounds in the ad.

For *characters*, the categories and values were

• Youth Actors: Do the ads feature kids in prominent roles?

• Adult Actors: Do the ads feature adults in prominent roles?

• Youth Actor Gender: The gender of prominent youth character(s)

• Adult Actor Gender: The gender of prominent adult character(s)

• Youth Character(s) Race: the race/ethnicity of prominent youth character(s)

• Adult Character(s) Race: the race/ethnicity of prominent adult character(s)

• Character(s) Gender: the gender of the majority of characters

• Character(s) Race/Ethnicity: the race/ethnicity of the majority of characters

The content index used in subsequent analyses was the linear sum of each of the content categories. Each ad received a content index score and this value was used in regression analyses as an independent variable as described below.

### Outcome measures used in this analysis

Our analysis focuses on the impact of parent reactions/receptivity on four primary measures of theorized cognitions related to parent-child communication: (1) social norms on waiting until older to have sex, (2) parent efficacy to talk to their child about sex, (3) short-term expectations about their child's response to parent communication about sex, and (4) long-term expectations about the impact of parent-child communication on their child's future success in life. Each outcome was measured as a multi-item summative scale or two-item index. We also examined the impact of ad strategies/features on parent reactions/receptivity to PSUNC ads. Parents' receptivity was also measured with a multi-item scale based on previous similar measures that have been tested in the health communications literature. These measures are described in more detail below.

#### Reactions/Receptivity to PSUNC Ads

For each ad shown in the survey, we asked a series of questions to measure parents' reactions and receptivity to those ads. These questions formed a 6-item scale of reactions/receptivity for each ad. The items included questions that assessed whether the ad: 1) was convincing; 2) grabbed the parent's attention; 3) gave the parent good reasons to talk to their kids about sex; 4) motivated parents to talk with friends or other adults family members about the ad; 5) motivated parents to talk with their child(ren) about the ad; and 6) the ad said something important to the parent. Each of these constructs was assessed with yes/no responses and then linearly summed form the reactions/receptivity scale for each ad.

#### Social norms for delay of sexual initiation

This measure included two items that asked participants their views regarding how long a child should wait before becoming sexually active; one item asked about boys, and the other asked about girls. Response options were "until they are 12," "until they are 14," "until they are 16," "until they are 18," "until they are 21," and "until they are married." Principal factor analyses indicated that both items loaded strongly onto a single factor (Eigen value = 1.69) with high reliability (alpha = 0.94).

#### Efficacy to talk to child about sex

We used four items to assess participants' belief in their ability to communicate with their child about sexual activity. Each item begins with the stem, "How sure are you that you can always explain to your child..." Seven category response options ranged from "completely sure" to "not at all sure" for each item. These items loaded strongly into a single factor (Eigen value = 1.81) with good reliability (alpha = 0.78).

#### Short-term outcome expectations

We had six items asking participants to consider the immediate impacts of parent-child communications about sex. Each item begins with the stem, "If you talked early and often with your child about sex, your child will..." and concludes with statements about the child's behavior (e.g., be less likely to be sexually active as a teen) and the child's perception of the parent (e.g., think you are a hypocrite). Four category response options ranged from "strongly agree" to "strongly disagree" with no neutral option. Principal factor analyses showed these items loaded into a single factor (Eigen value = 1.60) with acceptable reliability (alpha = 0.70).

#### Long-term outcome expectations

Two items ask participants to consider whether delaying sexual initiation would have a positive impact on their children's future. Four category response options ranged from "strongly agree" to "strongly disagree" with no neutral option. These two items loaded into a single factor (Eigen value = 0.83) with adequate reliability (alpha = 0.69).

The survey also included measures for a number of parent characteristics and other potential correlates of parent-child communication that we controlled for in our statistical analyses. These included parent marital status; educational attainment; race/ethnicity; age; full-time employment status; family structure (one or two parents in home); metropolitan statistical area (MSA) category (urban or rural); child gender; child's access to television, Internet, and other media in their bedroom; and an 8-item scale for parent involvement. The parent involvement scale includes items drawn from previous research that measure joint parent-child activities and the frequency of those activities [29,30]. The specific items in the parent involvement scale measure past month frequency ("never," "less often," "at least once a month," or "at least once a week") of (1) shopping, (2) going to the movies or sporting events, (3) watching television, (4) attending religious services, (5) doing homework, (6) attending a party, (7) volunteering, and (8) playing a game or a sport. Prior research shows that these items load into a single scale that has good reliability [4].

### Statistical Analysis

In a separate study conducted as part of the larger PSUNC evaluation, Davis and colleagues (2010) conducted principle factor analysis to assess reliability of each of the four cognitive outcomes examined in this study [5]. The factor analysis was performed using the principal factor method in Stata statistical software. Scale and index reliability were then estimated using Cronbach's alpha coefficient for the scale, a measure of the internal consistency of the scale determined by the average inter-item correlation and the number of items in the scale [31,32]. We estimated Cronbach's alpha overall and separately for mothers and fathers in the study. Principal factor analyses indicated that the items within each of the four primary cognitive outcome scales/indices that we examined load into a single scale or index with factor loadings ranging from 0.38 to 0.92 for each item and Cronbach's alphas ranging from 0.69 to 0.94 for each scale/index.

Additionally, as part of the current study, we conducted principle factor analysis to assess reliability of the reaction/receptivity scale items. The factor analysis was performed using the same procedure as above. Results are reported in Table 2 below.

**Table 2:** Factor Analysis Results

Message Reaction/Receptivity Scale Items	Factor Loadings	Chronbach's Alpha
This ad is convincing	.58	.86
Would you say the ad grabbed your attention?	.60	.85
Would you say the ad gave you good reasons to talk to your kids about sexual activity?	.57	.87
Did you talk to your friends or other adult family members (not your child[ren]) about this ad?	.73	.78
Did you talk to your child(ren) about the ad?	.71	.80
Would you say this ad said something important to you?	.64	.83

We conducted descriptive statistics of each of the control variables described earlier. All analyses were estimated using SAS (Statistical Analysis System, SAS Institution Inc., Cary, NC, USA) version 9.2.We used multivariable logistic regression procedures to construct separate models for each of the four PSUNC ads. We estimate the relation between the content index and reactions/receptivity for each PSUNC ads. All control variables described earlier were included in each model. The model outcome variables were dichotomous indicators for high or low reactions/receptivity scores, based on median split. Separate models were run for each ad.

We also used multivariable logistic regression to estimate the relationship between ad reactions/receptivity and the four cognitive outcome measures. As described earlier we constructed separate models for each of the fours PSUNC ads. All control variables described earlier were also included.

This study was approved by the Research Triangle Institute and George Washington University Institutional Review Boards (IRB) for human subjects protection.

## Results

### Descriptive characteristics of Sample

Briefly, as described earlier our sample consisted of parents recruited from the Knowledge Networks online panel. All respondents had children under age 18 living in the home, and 52.3% of these children were boys. Of these, 76.5% of them were White/Caucasian, 8.1% Non-Hispanic Black, and 8.0% Hispanic. Respondents were 81.5% married and 85.5% lived in a traditional 2-parent household. Of our respondents, 46.9% had completed a Bachelor's Degree or higher and 54.9% were employed full time, while 25.2% reported being unemployed. We primarily sampled urban and suburban respondents, with 87.5% reporting that they lived in these areas with the remainder living in rural settings. Thus, although the Knowledge Networks panel as a whole was sampled using a nationally representative methodology, our study sample was somewhat better educated, had higher rate of 2-parent households, and had higher percentage of White and lower percentages of Black and Hispanic respondents than the general US population.

### Factor analysis results

Table 2 shows results of the factor analysis used to create the reaction and receptivity (R/R) scale used in subsequent multivariable logistic regression models. All variables loaded on 1 factor at .575 or higher. Following the widely used Comrey & Lee (1992) criteria, we retained all items in the scale for subsequent analyses [33].

### Logistic regression models

Table 3 summarizes logistic regression models estimating the odds of high (positive) reaction/receptivity using the R/R scale developed through factor analysis given ad content. For this analysis, a higher content index represents the presence of more of the content features we coded for in the analysis, as described earlier. The analysis examines the R/R scale dichotomized using a median split to represent high versus low reaction/receptivity and recall for a specific ad. Higher odds ratios indicate higher a R/R score given the content associated with that ad resulting from our content analysis. None of the associations were significant for this analysis, indicating that the amount of ad content captured in our content analysis was not associated with differences in R/R score.

**Table 3:** Logistic regression of Message Receptivity on Content Index

Outcomes	OR	95% CI	p-value
R/R scale (Mimes)	0.989	(0.913, 1.071)	0.7832
R/R scale (Gadget)	0.881	(0.792, 0.980)	0.8708
R/R scale (Muffinhead)	0.990	(0.913, 1.073)	0.6948
R/R scale (Radio)	0.992	(0.913, 1.078)	0.7084

Note: Model controls for child gender; parent marital status; highest educational attainment; race/ethnicity; parent age; employment status; family structure; whether child had computer, cable television, or Internet in his/her bedroom; metropolitan statistical area urban status; parental involvement.

Table 4 summarizes logistic regression models estimating the odds of cognitive outcomes given dichotomous higher versus lower scores on the R/R scale. The R/R results were dichotomized using a median split to represent high versus low reaction/receptivity to a specific ad. Higher odds ratios indicate higher cognitive outcomes associated with an ad. We found that the gadget advertisement was consistently associated with higher odds of positive sexual communication cognitive outcomes on each of the 4 scales. We found a marginally significant (*p *< .10) negative association between the muffinhead advertisement and the long-term expectation scale, indicating a higher receptivity to Muffinhead was associated with *lower *long-term expectations. Finally, we found the radio ad was associated with slightly higher odds of positive short-term and long-term expectations.

**Table 4:** Logistic Regression of PSUNC Cognitive Outcomes by Advertisement on Message Receptivity (R/R scale)

Mimes advertisement			
Outcomes (Reference = Low)	OR	95% CI	p-value
Wait Until Older Norm Index	0.992	(0.782, 1.257)	0.9441
Efficacy Scale	1.073	(0.959, 1.199)	0.2181
Short-term Expectation Scale	1.008	(0.901, 1.128)	0.8898
Long-term Expectation Scale	1.063	(0.943, 1.197)	0.3178
**Gadget advertisement**			
**Wait Until Older Norm Index**	**2.749**	**(1.625, 4.649)**	**0.0002**
**Efficacy Scale**	**1.319**	**(1.039, 1.674)**	**0.0227**
**Short-term Expectation Scale**	**1.314**	**(1.032, 1.674)**	**0.0270**
**Long-term Expectation Scale**	**1.841**	**(1.426, 2.377)**	**<0.0001**
**Muffinhead advertisement**			
Wait Until Older Norm Index	1.017	(0.804, 1.287)	0.8859
Efficacy Scale	0.905	(0.809, 1.012)	0.0804
Short-term Expectation Scale	1.034	(0.923, 1.158)	0.5649
Long-term Expectation Scale	0.890	(0.789, 1.004)	0.0572
**Radio advertisement**			
Wait Until Older Norm Index	0.965	(0.748, 1.245)	0.7848
Efficacy Scale	0.898	(0.798, 1.011)	0.0753
**Short-term Expectation Scale**	**1.132**	**(1.005, 1.276)**	**0.0416**
**Long-term Expectation Scale**	**1.158**	**(1.019, 1.316)**	**0.0241**

Note: Model controls for child gender; parent marital status; highest educational attainment; race/ethnicity; parent age; employment status; family structure; whether child had computer, cable television, or Internet in his/her bedroom; metropolitan statistical area urban status; and parental involvement.

## Discussion

This study builds on previous research that examines the role of message content and message receptivity in behavioral determinants and behavior change. We found that message receptivity, but not message content characteristics, was associated with response to PSUNC, a large-scale sexual communication campaign. Message receptivity was most frequently associated with short-term and long-term expectations regarding the effectiveness of sexual communication in influencing children to wait before having sex, and one advertisement tested in our study, the Gadget ad, was found by far to be the most positively associated with message receptivity.

With respect to our first and second specific aims, we developed a detailed methodology for content analyzing sexual communication advertisements. We completed content analysis of 4 ads that were designed with differing message strategies and purposes in the PSUNC campaign based on an in-depth set of specific, measurable message and advertising execution features. We conducted coding using a formal content analysis procedure developed for the study based in part on previous research [9,10]. This study extends previous methods by examining features specific to parent-child sexual communication messaging, including active voice (parent versus child) and locus of control (parents lack control over their children) [34].

After developing the coding methodology, we developed quantitative measures of those features, including message strategies, marketing strategies, and voice and other stylistic features. We integrated those coded measures as variables in a larger person-ad dataset in order to evaluate the relationship between ad exposure, content, message receptivity, and attitudes & beliefs targeted by the PSUNC advertising. This approach extends previous research on message testing and effects of content on subsequent cognitive and behavioral changes by including new variables of interest [35-37]. This research also validates the use of the person-ad dataset methodology and presents an example of the use of this method to test a campaign theory of behavior change. We examined and validated the conceptual model, based on SCT, underlying PSUNC: Namely that high message receptivity to ads promoting specific campaign-targeted attitudes and beliefs are associated with observed changes in those outcomes [4,21]. We observed increases in all 4 targeted attitudes and beliefs - norms, self-efficacy, short- and long-term expectations - for the Gagdet ad, and increases in short- and long-term expectations for the radio ad.

Regarding our third aim, we evaluated the relationship between message content, as captured by our coding scheme, and message receptivity, and then the relationships between message receptivity and parents' cognitions related to sexual communication. With regard to the first relationship, content and receptivity, we observed no significant effects. Content features of ads, as captured in our content index, were not associated with message receptivity.

There are several possible explanations for this result. One is that the stylistic features of these ads were subordinate to the salience of the issue of children, sex, and parent-child communication. Parents may have focused simply on the message itself and not on stylistic and other features captured in our content index. Another explanation is that the ads may have lacked sufficient variation in the content features we measured to detect an effect on message receptivity. The ads may simply not have had sufficient sensation value, production quality, distinctive voice or other features. Finally, some third ad feature or set of features not measured in our study may be associated with receptivity to sexual communication messages. Future research should examine these hypotheses.

However, respondents were highly receptive to the messages in the Gadget ad and receptivity to the ad was associated with positive effects on all 4 targeted sexual communication attitudes and beliefs. Gadget appears to be a highly effective ad that produces outcomes targeted by the campaign. Additionally, the radio ad was effective, although with much smaller effects, in increasing short-term and long-term expectations for the effectiveness of parent-child communications. This follows from SCT and the PSUNC conceptual model, and changes in these attitudes have been shown to predict subsequent increased parental communications about sex [4,7].

Thus this study provides additional evidence to support the PSUNC conceptual model: Exposure to PSUNC produces (in case of some ads) high message receptivity, which in turn produces increases in targeted attitudes and beliefs about the norms, benefits, and outcomes of parent-child sexual communication, which in turn produces increased communication.

Additionally, this study demonstrates the value of message receptivity as a measure of campaign response. Message receptivity has predictive value as it is associated with positive attitudes and beliefs targeted by campaigns such as PSUNC. Message receptivity is an important measure to use in pre-testing campaign messages during formative development, and to evaluate the mechanisms by which campaign affects cognitive outcomes after implementation.

Several limitations should be noted. First, the study is cross-sectional: We examined data from one wave of the PSUNC media tracking survey. Thus no causal inferences can be made, although it should be noted that other published studies have demonstrated a causal effect of PSUNC exposure consistent with the present study [4,7]. Second, while our sample was nationally representative, it was somewhat more White, better educated, and had higher proportion of 2-parent households than the US population. We controlled for these variables in analysis, but readers should use caution in generalizing to the general population. Finally, there are inherent limitations in a content analysis methodology as compared to primary data collected on content. We followed a rigorous content review and analysis methodology, but reviewer bias is possible in any such exercise. Future studies should collect primary data on message content from respondents who have viewed the content as a preferred methodology.

## Conclusion

Message content and receptivity are important variables to use in evaluation public health campaigns. Future studies should continue to develop these measure and analysis methods. PSUNC is an effective campaign to promote parent-child communication. Following the campaign's conceptual model, message receptivity partially explains observed increases in targeted campaign attitudes and beliefs among respondents exposed to the ads. In particular, the Gadget and radio ads were effective in promoting targeted attitudes and beliefs. Future parent-child sexual communication campaigns should use these findings in developing message and advertising executions.

## Competing interests

The authors declare that they have no competing interests.

## Authors' contributions

WDE conceptualized the study, developed the analysis plan, supervised analysis, and wrote sections of the paper. KCD contributed to the analysis plan, assisted in the analysis, and wrote sections of the paper. CU and KP coded and analyzed PSUNC ad content. MK conducted statistical analysis and wrote sections of the paper. All authors read and approved the final manuscript.
